# Are Environmental volatiles contributing to Insecticide resistance in Anopheles Mosquitoes in Ghana?

**DOI:** 10.21203/rs.3.rs-6606369/v1

**Published:** 2025-05-16

**Authors:** Christopher Mfum Owusu-Asenso, Isaac Kwame Sraku, Nana Aba Setorwu Eyeson, Anisa Abdulai, Abdul Rahim Mohammed Sabtiu, Simon Kwaku Attah, Fred Aboagye-Antwi, Yaw Asare Afrane

**Affiliations:** Centre for Vector-Borne Disease Research, Department of Medical Microbiology, University of Ghana Medical School; Centre for Vector-Borne Disease Research, Department of Medical Microbiology, University of Ghana Medical School; Centre for Vector-Borne Disease Research, Department of Medical Microbiology, University of Ghana Medical School; Centre for Vector-Borne Disease Research, Department of Medical Microbiology, University of Ghana Medical School; Centre for Vector-Borne Disease Research, Department of Medical Microbiology, University of Ghana Medical School; Centre for Vector-Borne Disease Research, Department of Medical Microbiology, University of Ghana Medical School; African Regional Postgraduate Programme in Insect Science, Department of Animal Biology and Conservation Science, College of Basic and Applied Sciences, University of Ghana; Centre for Vector-Borne Disease Research, Department of Medical Microbiology, University of Ghana Medical School

**Keywords:** Insecticide resistance, heavy metals, pesticide residues, Anopheles gambiae s.l., kdr mutations, mining activities

## Abstract

Insecticide-based interventions such as IRS and LLINs have significantly reduced malaria transmission globally. However, their sustainability is increasingly threatened by insecticide resistance. While insecticide and pesticide use are known resistance drivers, the role of environmental volatiles remains underexplored. This study investigated the impact of environmental volatiles on insecticide resistance by sampling *Anopheles* larvae from six sites in Ghana, including petroleum spill sites, mining areas and industrial zones. WHO bioassays revealed resistance to clothianidin (54% – 80%) and chlorfenapyr (80% − 84%) all site categories. Interestingly, high-intensity resistance to pirimiphos methyl (10x = 1.7%) was detected in vectors from Obuasi. High-intensity pyrethroid resistance [deltamethrin (10x = 79–92%); permethrin (10x = 74–95%)] was observed across all sites, with varying frequencies of *kdr* mutations (*L995F, V402L, I1527T, P1874L*; 0.12–0.98) were observed across all sites. High frequency of Ace-1 (0.62) was observed in Obuasi. Chemical analyses of water from breeding habitats revealed significant associations between heavy metals and insecticide resistance (*P* = 0.04). *Anopheles coluzzii* (81.7%) was the dominant species across all sites. These findings provide evidence that environmental volatiles may contribute to insecticide resistance. There is an urgent need for enhanced surveillance and resistance management strategies for effective malaria vector control.

## Background

Globally, substantial reductions in malaria morbidity and mortality have been achieved using insecticide-based interventions^1, 2, 3^. However, the sustainability of these vector control interventions is threatened by a surge in insecticide resistance in *Anopheles* mosquito populations, which poses a significant challenge to the effectiveness of these interventions^4^. The continuous use of insecticides for public health and pesticides in agriculture^5, 6^ have been implicated in driving selection for insecticide resistance in malaria vectors.

Recent evidence suggests that the discharge of industrial effluents, and heavy metals from mining activities^7^ into mosquito breeding habitats either directly or through runoff may contribute to the development of insecticide resistance in malaria vectors. Contamination from petroleum products (oil and fuel spills)^8, 9^ and heavy metals such as cadmium (Cd), copper (Cu), lead (Pb), mercury (Hg), arsenic (As), and zinc (Zn)^10, 11, 12^ can degrade water quality and create toxic conditions that impose selective pressures on mosquito larvae. Prolonged exposure to these pollutants may induce physiological stress responses, leading to metabolic adaptations that enhance insecticide tolerance and drive resistance evolution in mosquito populations^8, 9, 13, 14, 15^. Furthermore, complex interactions between petroleum pollutants and insecticide targets may facilitate cross-resistance mechanisms in malaria vectors, intensifying the challenge of vector control^8, 14^.

Additionally, rapid urbanization and industrial expansion across sub-Saharan Africa are introducing selective pressures on malaria vectors through the release of effluents into drainage systems where mosquitoes breed. *Anopheles* mosquitoes have demonstrated an ability to thrive in polluted habitats, where exposure to household and industrial contaminants may drive insecticide resistance. Organic pollutants can upregulate detoxification enzymes such as cytochrome P450s, GSTs, and esterases in *Anopheles* mosquitoes, enhancing insecticide tolerance and reducing the efficacy of chemical control measures^16^.

The presence of environmental volatiles in mosquito breeding habitats as a result of petroleum spillage, mining activities, and industrial effluents may present a novel avenue for understanding how these volatiles may mediate insecticide resistance in *Anopheles* vector populations. It is hypothesized that the chemical compounds released from these environmental volatiles may bear structural similarities to those used in insecticides for vector control, and may also have the potential to disrupt the physiological pathways of these mosquitoes. Consequently, exposure of mosquito larvae to sublethal doses of these compounds may ultimately trigger a selection process in mosquitoes breeding in these habitats, influencing their susceptibility to insecticides commonly employed in vector control programmes.

This study aimed to investigate how volatiles, including heavy metals, and environmental contaminants from mining activities, petroleum-derived compounds and industrial effusions, contribute to the development of insecticide resistance in malaria vectors. Furthermore, elucidating the association between these volatiles and insecticide resistance in malaria vectors will provide insights into their contribution to the increasing levels of insecticide resistance.

## Results

### Phenotypic Resistance Profile of An. gambiae s.l. Using WHO Tube Bioassay

A total of 2,700 female *Anopheles* mosquitoes (n = 1,800 exposed and n = 900 control) were phenotyped. Insecticide susceptibility profiles of *An. gambiae* s.l. populations across the four site categories displayed varying resistance levels to the three insecticides tested. Vector population from petroleum-use sites demonstrated high-intensity resistance to deltamethrin in Abossey Okai (1x = 20%, 5x = 34%, 10x = 75%); Kokompe (1x = 4%, 5x = 33%, 10x = 66%); and Tamale Fitam (1x = 35%, 5x = 62%, 10x = 78%). High-intensity resistance to permethrin was observed across all the study sites in the petroleum sites, (Fig. 2a). *Anopheles* population from Abossey Okai and Kokompe displayed partial resistance to pirimiphos-methyl (1x = 92% − 94%) but reached full susceptibility (100%) after exposure to 5x concentration of the insecticide, indicating a low-intensity resistance (Fig. 2a).

*Anopheles* mosquitoes from the mining site category (Obuasi) showed extremely high-intensity resistance to pyrethroids [deltamethrin (1x = 4%, 5x = 53%, 10x = 79%); permethrin (1x = 5%, 5x = 11%, 10x = 74%)] and pirimiphos-methyl (1x = 0%, 5x = 1.7%, 10x = 1.7%), (Fig. 2b). Vector population from the industrial sites [Tema community 1 (1x = 13%, 5x = 81%, 10x = 90%; Accra Industrial area (1x = 56%, 5x = 79%, 10x = 92%)] showed a high-intensity resistance to deltamethrin. High-intensity resistance to permethrin was observed across all the study sites in the petroleum sites category, (Fig. 2c). Vector populations from the Industrial sites category were susceptible to pirimiphos-methyl, (Fig. 2c).

### Phenotypic Resistance Profile of An. gambiae s.l. to Chlorfenapyr and Clothianidin Using WHO Bottle Bioassay

A total of 1,800 female *An. gambiae* s.l. (n = 1,200 exposed and n = 600 control) were used to test for chlorfenapyr 100 μg/bottle and clothianidin 4 μg/bottle. Resistance to chlorfenapyr was observed in *Anopheles* vectors in Obuasi (mining site category) (95% − 97%) and Abossey Okai (petroleum sites category) (80% – 84%) after 72 h post-exposure to the insecticide (Fig. 3). However, complete susceptibility (100%) was observed in mosquitoes from Kokompe and Tamale Fitam after 24hrs. Complete mortality (100%) within 24 h was observed with vectors from Tema community 1 and Accra industrial area (industrial sites category) (Fig. 3).

Resistance to clothianidin was observed in mosquitoes from Obuasi (80%) and Tema Community 1 (54%) after 24 h post-insecticide exposure. Partial resistance after 24 h post-exposure was observed in *Anopheles* mosquitoes from Abossey Okai (95%) and Kokompe (94%). However, full susceptibility was observed in *Anopheles* populations from Tamale Fitam (petroleum sites category) and Accra Industrial area (industrial sites category) (Fig. 4). The reference strain (Kisumu susceptible strain) exhibited susceptibility to all insecticides, demonstrating 100% mortality at WHO-recommended discriminating dosages, thereby validating the quality of the insecticides used in coating the bottles.

### kdr Gene Mutations Detection in An. gambiae s.l.

*Anopheles* mosquitoes from all site categories (mining, industrial and petroleum) harboured high frequencies of *L995F* (0.82–0.98) and *P1874L* (0.65–0.77), (Table 2). *V402L* (generated by either of two nucleotide variants: *402L*(C) and *402L*(T)) and *1527T* which are completely linked had a frequency of (0.73–0.92) and (0.52–0.65) respectively. The *N1570Y* and *L995S kdr* genes were present at low frequencies of (0.15–0.57) and (0.12–0.37) respectively, across all sites (Table 1).

The *Ace-1*^*R*^ *G280S* mutation which is associated with pirimiphos-methyl resistance was significantly associated with the site category (*χ*^2^ = 74.82, df = 6, *P* = < 0.001) with relatively high allelic frequencies observed in *Anopheles* mosquitoes from Obuasi (0.62) (Table 1).

### Species Discrimination of Anopheles gambiae s.l.

A sub-sample of randomly selected *An. gambiae* s.l. (n = 240 mosquitoes; 40 per each study site) were used for sibling species discrimination Results from PCR assay revealed that the most abundant species was *An. coluzzii* (81.7%), followed by *An. gambaie* s.s. (7.5%), *An. arabiensis* (7.1%) and hybrid (3.8%). *Anopheles coluzzii* was the most abundant species at all sites (*χ*^2^ = 36.9, df = 12, *P* < 0.001). *Anopheles arabiensis* was detected only in Tamale Fitam (petroleum sites category) (42.5%) (Table 2).

#### Chemical Analysis of Water Samples from Breeding Habitats

Chemicals such as pesticide residues present in water samples collected from mosquito breeding habitats were identified using GC-MS. Four different chemicals including insecticides, herbicides and fungicides were detected above the level of quantification (LOQ; 0.05 μg/L) at the various study sites. In Abossey Okai, (petroleum site category), clothianidin (CTD) a neonicotinoid was detected in water samples from breeding habitats at a concentration of 0.054 μg/L. In Tema, imidacloprid (IMI) (neonicotinoid) and metalaxyl (MTL) a fungicide, were detected at concentrations of 0.064 μg/L and 0.12 μg/L, respectively. Atrazine (ATR) a fungicide, was also detected at concentrations of 0.13 μg/L in Accra industrial area (Table 3).

### Heavy Metal Detection in Anopheles Breeding Habitats

The analysis of larval breeding water for the presence of heavy metals across different site categories revealed significant contamination, particularly in mining, industrial, and petroleum-impacted areas. In Obuasi (mining site category), manganese (Mn) levels reached a notable 1.05 ppm, significantly exceeding the standard reference value of 0.04 ppm. Additionally, high levels of zinc (Zn) and strontium (Sr) were recorded at 0.50 ppm and 0.19 respectively (Table 4). In Abossey Okai (petroleum site), Sr concentrations (0.69 ppm) were particularly high, with Mn and Zn concentration levels detected at 0.84 ppm and 0.23 ppm, respectively. The presence of copper (Cu) in Abossey Okai was slightly elevated at 0.06 ppm, along with Ti detected at 0.17 ppm and lead (Pb) at 0.04 ppm. Water samples from Tema Community 1 (Industrial sites category) had high levels of Mn (0.22 ppm), Zn (0.10 ppm) and Sn (0.56 ppm) (Table 4).

### Association Between Heavy Metals and Insecticide Resistance in An. gambiae s.l.

A logistic regression analysis revealed a significant relationship between heavy metal concentrations and insecticide resistance in *Anopheles* mosquitoes to three insecticides: chlorfenapyr, clothianidin, and pirimiphos-methyl, (Table 5). A unit increase in Mn concentration was associated with a 6.85-unit increase in the log-odds of resistance to chlorfenapyr (95% CI: 3.22–10.49, *P* = 0.006). A unit increase in Sr corresponded to a non-significant 2.24-unit increase in the log-odds of chlorfenapyr resistance in *Anopheles* mosquitoes (95% CI: −3.49–7.96, *P* = 0.339). Similarly, a unit increase in Barium (Ba) was associated with a −0.93-unit change in the log-odds of resistance to chlorfenapyr (95% CI: −6.09–4.24, *P* = 0.664), (Table 5).

Furthermore, a unit increase in Pb was associated with a substantial 120.44-unit increase in the log-odds of clothianidin resistance (95% CI: 11.39–229.49, *P* = 0.036), whilst Copper (Cu) showed a 144.60-unit increase in clothianidin resistance (95% CI: 66.86–222.33, *P* = 0.007). Moreover, a unit increase in Ti resulted in a 45.61-unit increase in the log-odds of clothianidin resistance (95% CI: 3.67–87.54, *P* = 0.038). However, no significant associations were observed with any of the tested heavy metals to pirimiphos-methyl resistance, (Table 5).

## Discussion

Insecticide resistance in malaria vectors poses a significant challenge to malaria control programmes. Elucidating the underlying mechanisms and environmental factors contributing to resistance is crucial for developing specific and sustainable vector management strategies. This study investigated how some volatiles in the environment may mediate resistance in *Anopheles* mosquitoes in Ghana, with a focus on sites where environmental volatiles such as petroleum spillage, industrial effluents, and mining chemicals are present. The study findings indicate the heterogeneity in insecticide resistance profiles of *Anopheles* mosquitoes and the significant association of heavy metals with insecticide resistance in *An. gambiae* s.l..

Phenotypic resistance to chlorfenapyr and clothianidin, insecticides recently introduced in next-generation bednets and IRS respectively, was observed in mosquito populations from all site categories. This is a major public health concern, as these novel insecticides were specifically deployed to manage widespread resistance to pyrethroids and organophosphate in malaria vectors. High-intensity resistance to pyrethroids was also observed across all study sites, with low mortality rates even at 10× diagnostic doses. Moreover, genotypic analysis, which revealed high frequencies of *kdr* mutations (*L995F, V402L, I1527T, P1874L*) in *Anopheles gambiae* s.l. across all sites, aligns with the phenotypic resistance patterns observed in this study.

A key factor contributing to the observed resistance patterns could be the presence of chemical residues (clothianidin, imidacloprid, metalaxyl, and atrazine) and heavy metals such as Ti, Sr, Pb, As, Zn, Ba, and Mn in mosquito breeding habitats^7, 8, 17, 18^. These insecticides and agrochemicals, long used in agriculture, may have mediated resistance through continuous exposure^19, 20^. Their detection in larval habitats suggests, they may impose strong selection pressure on mosquito larvae, either by inducing detoxification pathways or physiological stress^11, 12, 20, 21^. These findings corroborate with findings from other studies that reported increased resistance to pyrrole and neonicotinoids due to high pesticide usage and other environmental stressors^22, 23^, which may drive the emergence of cross-resistance across multiple insecticide classes. Other studies have also reported increased insecticide resistance and high frequencies of *kdr* mutations^7, 8, 11^ in mosquitoes associated with environmental contamination from oil products, industrial effluents^24, 25^, and agricultural chemicals^26, 27^.

An extremely high-intensity resistance to pirimiphos methyl was observed in *Anopheles gambiae* s.l. population in Obuasi, a site heavily impacted by mining activities. However, partial resistance to pirimiphos-methyl was detected in mosquito populations from petroleum-contaminated sites, while vectors from other areas remained fully susceptible. The extremely resistant phenotype observed in *Anopheles* mosquitoes from Obuasi could be attributed to the continuous exposure of *Anopheles* mosquitoes to heavy metals such as Manganese, Zinc, Barium, Strontium, Boron, Titanium, and Chromium, released directly into mosquito breeding habitats or through mining-related run-off^7, 13, 17, 28^. Moreover, the alarming level of resistance to pirimiphos-methyl observed in this site is likely a direct consequence of sustained IRS campaigns conducted from 2006 to 2024, with pirimiphos-methyl specifically used extensively between 2006 to 2017. This prolonged and repeated exposure may have exerted intense selection pressure on local mosquito populations, leading to the extremely high-resistance intensity observed in this study.

## Conclusion

This study’s findings demonstrate a significant association between environmental volatiles and insecticide resistance development in *An. gambiae* s.l. The extremely high levels of resistance observed are particularly alarming and raise serious concerns about the sustainability of current vector control strategies. These results emphasizes the critical need for continuous surveillance and improved resistance management strategies to ensure effective vector control.

## Materials and Methods

### Study Sites

**Figure 1:**
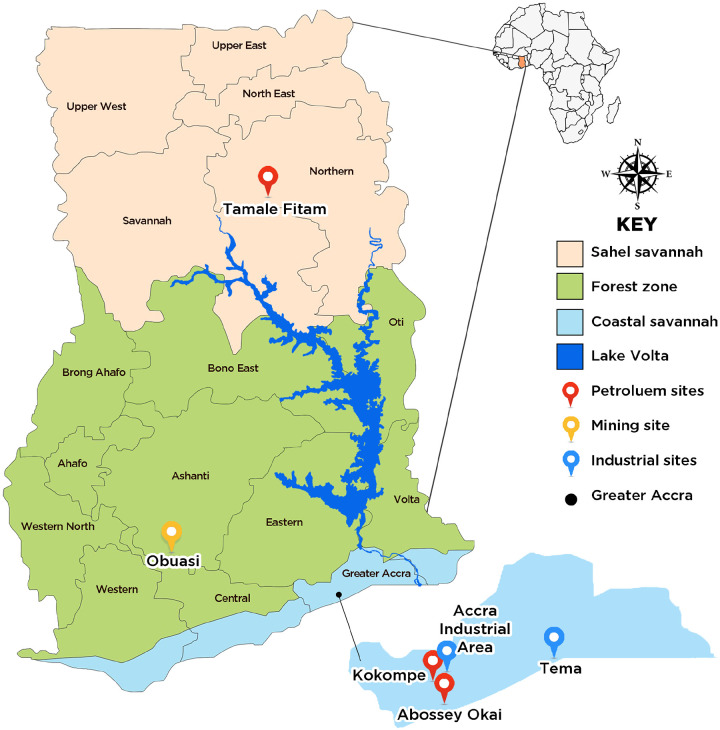
Map of Ghana showing the various study sites

**Figure 2:**
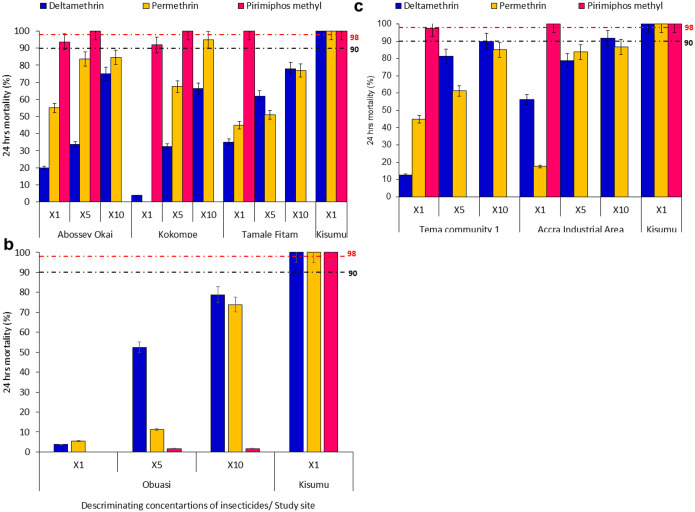
WHO Intensity Bioassays Test Results on Mosquitoes from a: Petroleum Sites, b: Mining site, c: Industrial sites. Error bars indicate a 95% confidence interval. The WHO criteria specify a 90% mortality threshold to indicate suspected resistance and a 98% threshold to confirm susceptibility with black and red dotted lines respectively.

**Figure 3:**
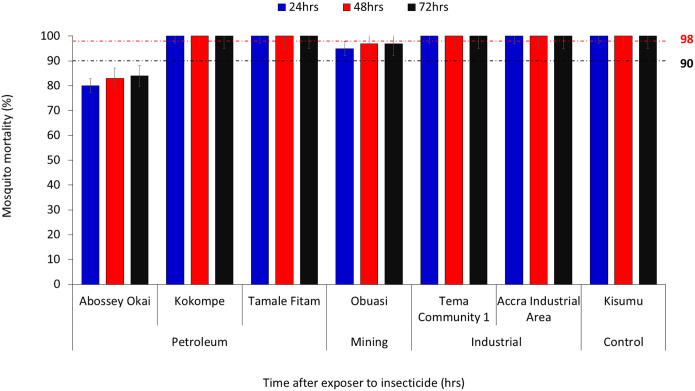
WHO Bottle Bioassay Test Results Using Chlorfenapyr for the Different Sites. Error bars indicate a 95% confidence interval. The WHO criteria specify a 90% mortality threshold to indicate suspected resistance and a 98% threshold to confirm susceptibility with black and red dotted lines respectively.

**Figure 4:**
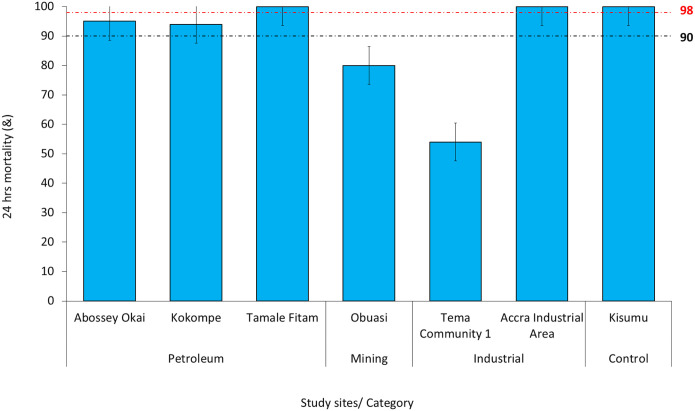
WHO Bottle Bioassay Test Results Using Clothianidin for the Different Sites. Error bars indicate a 95% confidence interval. The WHO criteria specify a 90% mortality threshold to indicate suspected resistance and a 98% threshold to confirm susceptibility with black and red dotted lines respectively.

This study was conducted across six sites in Ghana, selected to represent diverse ecological zones and environmental stressors that could facilitate the mediation of insecticide resistance in *Anopheles* mosquitoes (Fig. 1). These sites were categorized into informal vehicle repairing sites where petroleum gets spilt through the repair of cars (herein after: petroleum sites category) which were: Abossey Okai, Kokompe, Tamale Fitam; sites where surface mining is undertaken (mining site category: Obuasi) and sites with industries where effluents are spilt in the environment (Industrial sites category: Tema community 1, Accra Industrial Area).

Abossey Okai (5.5480° N, 0.2424° W) and Kokompe (5.5813° N, 0.2173° W) are both located in the city of Accra, in the coastal savannah zone of Southern Ghana. Oil spills as a result of engine oil changes and vehicle repairs, and the leaching of metal compounds into mosquito breeding habitats in these sites, may trigger an adaptive response and increase resistance in the vectors. Tamale Fitam (9.4198° N, 0.8199° W), located in the Sahel-savannah zone of Northern Ghana, may also have oil spills as a result of automobile repair activities similar to Abossey Okai and Kokompe.

Obuasi (6.2024° N, 1.6658° W), located in Ghana’s middle forest zone, is known for its intensive deep and surface mining for decades. These mining operations create pockets of breeding habitats for *Anopheles* mosquitoes. Processing the gold ore with mercury, zinc, cyanide and other heavy metals introduces toxic volatiles into the environment, which may leach into larval habitats and exert chronic selection pressure on mosquito populations. Moreover, there has been a prolonged IRS campaign by the AngloGold Ghana malaria control program, spanning nearly two decades (2006–2024), during which mosquito populations in Obuasi have been exposed to successions of different insecticide classes. These include organophosphates and carbamates (e.g., pirimiphos-methyl, extensively used from 2006–2017), pyrethroids, neonicotinoids, neonicotinoid + pyrethroid combinations (2018–2023), and more recently, meta-diamides. The sequential and overlapping intense selection pressures from both environmental contaminants and long-term insecticide use may be key mediators of insecticide resistance in *Anopheles gambiae* s.l.

Tema Community 1 (5.6698° N, 0.0200° W), located in the coastal savannah of southern Ghana, is an industrial and port city. The dense concentration of manufacturing facilities and port operations generates significant industrial effluents, many of which are discharged into open drains and stagnant pools that double as mosquito breeding grounds. Similarly, Accra Industrial Area (5°33’16.992” N, 0°13’15.492” W), located in the city of Accra, is a densely populated industrial zone characterized by a high concentration of manufacturing facilities. These factories are potential sources of atmospheric pollutants and chemical effluents, which often discharge into open drains and stagnant water bodies. the presence of these pollutants in drainages where mosquitoes could be breeding may exert selection pressures capable of inducing or enhancing insecticide resistance in local *Anopheles* vector populations.

The coastal savannah in southern Ghana has a tropical savannah climate with temperatures ranging from 23–34°C and an average annual rainfall of 787 mm, following a bimodal pattern with rainy seasons from April–June and October–November. The dry season lasts from December to March. The forest zone in the middle of Ghana experiences a tropical forest climate with 1,500–2,000 mm of annual rainfall, also in a bimodal pattern, with rainy seasons from March–July and September–November, and dry periods from August–February. Temperatures remain stable between 24–30°C. The Sahel savannah in the north of Ghana has a unimodal rainfall pattern from May–November, averaging 900 mm annually. The dry season (December–April) sees temperatures rising to 42°C, with a mean annual temperature of 28°C.

### Mosquito Larval Collection and Raising in the Insectary

*Anopheles* larvae sampling was carried out from January 2023 to July 2024. To avoid the collection of sibling species, larvae were sampled randomly from different breeding habitats in each study site. This study was approved by the Ethics and Protocol Review Committee of the College of Health Sciences, University of Ghana (protocol identification number: CHS-Et/M.8-P4.6/2023–2024). All methods were carried out in accordance with relevant guidelines and regulations. Meetings were held at each study site with chiefs, community leaders, and residents to introduce the research. Permission to conduct the study at the various sites was obtained from community leaders. Verbal informed consent was obtained from community leaders and residents for mosquito sampling activities.

Immature *Anopheles* mosquitoes collected from the same site within the Sahel-savannah zone were transported to the insectary of the President’s Malaria Initiative (PMI) Project Office in Tamale. Similarly, larvae collected from sites within the forest and coastal savannah ecozones were transported to the insectary of the AngloGold Ashanti malaria control programme (AGAmal) in Obuasi, and the Department of Medical Microbiology, University of Ghana Medical School, Korle-Bu, Accra, respectively. The mosquito larvae were raised to adults in the insectary, maintained at an average temperature of 28 ± 1°C and a relative humidity of 80.9 ± 6.3%. Once emerged, the adult mosquitoes were provided with a 10% sucrose solution for feeding. Three to five (3–5) days old sugar-fed, but not blood-fed, females were later selected for WHO susceptibility tests and intensity bioassays.

#### Phenotypic Resistance in Anopheles Mosquitoes Using WHO Susceptibility Tube Bioassay

To assess the resistance intensity of *An. gambiae* s.l. population across various site categories, a batch of 25 non-blood-fed females that were 3–5 days old were subjected to the WHO susceptibility test bioassay. Four replicates and 2 controls were used for each insecticide using the standard WHO tube assay procedure^29, 30^. The WHO test papers impregnated with insecticides at discriminating concentrations used were: permethrin [1x (0.75%), 5x (3.75%), and 10x (7.5%)], deltamethrin [1x (0.05%), 5x (0.25%), and 10x (0.5%)], and pirimiphos-methyl [1x (0.25%), 5x (1.25%), and 10x (2.5%)]^30^. The insecticide papers were initially tested on the Kisumu susceptible strain, a reference strain susceptible to insecticides, to confirm their efficacy.

#### Phenotypic Resistance to Chlorfenapyr and Clothianidin in Anopheles gambiae s.l. using WHO Susceptibility Bottle Bioassay

To determine the susceptibility status of *Anopheles gambiae* s.l. to chlorfenapyr and clothianidin, the WHO Bottle bioassays were carried out using four replicates according to WHO guidelines^31^; each replicate consisted of 25 female *Anopheles* mosquitoes aged 3–5 days. These mosquitoes were exposed to 100 μg/ml chlorfenapyr-acetone solution and 4 μg/ml clothianidin-acetone-MERO solution for 1 hour; this was done approximately 24 h after coating the bottles with the respective insecticides. Two additional replicates of 25 mosquitoes each served as a negative control, with bottles treated with 1 ml of acetone for chlorfenapyr, or acetone with MERO for clothianidin. The Knocked-down mosquitoes were recorded at the end of the 60 min exposure period. After exposure, mosquitoes were transferred to a paper cup covered with untreated netting and provided with a 10% sugar solution soaked in a wad of cotton, which was changed daily; the assay was monitored and mortalities were recorded at 24 hrs, 48 hrs, and 72 hrs. The Kisumu susceptible strain, maintained under the same insectary conditions, was utilized as the primary test control to monitor the quality of the coated bottles.

#### Screening for Heavy Metals and Insecticide Residues in Water Samples from Anopheles Breeding Habitats

Water samples were collected from selected breeding sites categorized into the three distinct site categories: mining, industrial and petroleum sites. Water samples (50 ml each) were collected and prepared in triplicate for chemical residue analysis. These samples were transported to the Ghana Standards Authority (GSA) laboratory in Accra and analysed using high-performance gas chromatography and mass spectrometry (GC-MS), applying multi-residue analysis methods, with a detection limit of 10 ppb^32^ to determine the presence and concentration of pesticides and insecticides.

In addition to the insecticide residue analysis, the same water samples were screened for heavy metals at the GSA laboratory. This analysis was performed using a Perkin Elmer Nexion 2000 Inductively Coupled Plasma Mass Spectrometer equipped with a micro-mist nebulizer, quartz spray chamber and Peltier cooler according to the Environmental Protection Agency (EPA) 200.8 method for total recoverable metals^32^.

#### Detection of Target-Site Mutations in An. gambiae s.l.

Conventional and real-time PCR were used to investigate the presence of insecticide resistance genes, including *kdr haplotypes* (*L995F, L995S, N1570Y, V402L, I1527T, and P1874L*) and *Ace-1*^*R*^ *G280S*^33^. The allele-specific PCR procedure for *kdr* genotyping was used to detect *kdr* alleles using the protocol and primer sequences described by Martines-Torres *et al*. and Jones *et al*.^34, 35^ for *N1570Y*; Williams *et al*.^36^ for *V402L*, I1527T and *P1874L*; and Weil *et al*.^37^ for the *Ace-1*^*R*^ *G280S* mutation.

#### Characterization of Anopheles gambiae s.l.

A sub-sample of the mosquitoes after the phenotypic susceptibility testing were randomly selected and identified morphologically using the keys of Gillies and Coetzee^38^. Members of the *An. gambiae* s.l. were further identified by PCR to distinguish sibling species using a leg of each individual mosquitoes, as previously described by Scott et al.^39^.

### Data Management and Analysis

Descriptive analysis was done to visualize WHO susceptibility data, resistant allele frequencies, and mosquito species composition from the selected sites using graphs and tables.

WHO insecticide susceptibility levels were classified using the WHO criteria^31, 40^. Allele frequencies of resistance gene markers in the vector populations at each site were calculated using Hardy-Weinberg equilibrium (HWE), with the formula F (allele frequency) = (2nRR + nRS) / 2N. Logistic regression was used to assess the association between insecticide resistance with categorical data and the presence of heavy metals. The concentration and identification of pesticide residue compounds and heavy metals were determined using reference standards (commercial), relative retention times and mass-to-charge ratios (GS-MS, model QP2010). P ≤ 0.05 was considered significant. All statistical analyses were done in R 4.2.2 via RStudio (2022.12.0 + 353) and STATA/IC 14.1.

## Figures and Tables

**Table 1:** Allele Frequency Distribution of Resistant Gene Markers of *An. gambiae* s.l.

Study site	Category	N	Resistant gene markers (F)						
			*N1570Y*	*V402L*	*I1527T*	*L995F*	*L995S*	*G208S*	*P1874L*
Tamale Fitam	Petroleum	30	0.27	0.92	0.55	0.93	0.35	0.07	0.73
Abossey Okai		30	0.42	0.73	0.52	0.97	0.33	0.13	0.75
Kokompe		30	0.15	0.87	0.57	0.98	0.12	0.08	0.77
Obuasi	Mining	30	0.57	0.87	0.60	0.98	0.27	0.62	0.70
Tema community 1	Industry	30	0.42	0.85	0.64	0.95	0.37	0.17	0.68
Accra Industrial Area		30	0.48	0.88	0.65	0.82	0.20	0.23	0.65

F = allele frequency, N = number of samples genotyped

**Table 2:** Species Discrimination of *An. gambiae* s.l.

Species	Petroleum			Mining	Industrial		Total (%)
	Tamale Fitam N (%)	Abossey Okai N (%)	Kokompe N (%)	Obuasi N (%)	Tema Community 1 N (%)	Accra Industrial Area N (%)	
*An. gambiae* s.s.	11 (27.5)	0 (0.0)	5 (12.5)	1 (2.5)	0 (0.0)	1 (2.5)	18 (7.5)
*An. coluzzii*	9 (22.5)	40 (100.0)	33 (82.5)	38 (95.0)	40 (100.0)	36 (90.0)	196 (81.7)
*An. arabiensis*	17 (42.5)	0 (0.0)	0 (0.0)	0 (0.0)	0 (0.0)	0 (0.0)	17 (7.1)
Hybrid	3 (7.5)	0 (0.0)	2 (5.0)	1 2.50)	0 (0.0)	3 (7.5)	9 (3.8)
**Total**	**40**	**40**	**40**	**40**	**40**	**40**	**240 (100.0)**

**Table 3:** Chemical analysis, stratified by study site and site category

Study sites	Category	ATR (μg/L)	CTD (μg/L)	IMI (μg/L)	MTL (μg/L)	LOQ(μg/L)
Abossey Okai		bt	0.054	bt	bt	0.05
Kokompe	Petroleum	bt	bt	bt	bt	0.05
Tema Community 1		bt	bt	0.064	0.12	0.05
Accra Industrial Area	Industrial	0.13	bt	bt	bt	0.05
Obuasi	Mining	bt	bt	bt	bt	0.05

ATR = Atrazine, CTD = Clothianidin, IMI = Imidacloprid, MTL = Metalaxyl, LOQ: Level of quantification, bt: below threshold of 0.05μg/L

**Table 4:** Comparative Analysis of Heavy Metal Contamination in Mosquito Breeding Habitat across Different Site

Study site	Site category	B 11 (ppm)	Ba-1 137 (ppm)	Cr-1 52 (ppm)	Cu 63 (ppm)	Mn 55 (ppm)	Pb 208 (ppm)	Sr 88 (ppm)	Ti 47 (ppm)	Zn 66 (ppm)
Abossey Okai	Petroleum	0.20	0.33	0.02	0.06	0.84	0.04	0.69	0.17	0.23
Kokompe	Petroleum	0.06	0.17	0.01	0.02	0.10	0.01	0.21	0.02	0.08
Obuasi	Mining	0.10	0.17	0.06	0.03	1.05	0.02	0.19	0.07	0.50
Accra Industrial Area	Industrial	0.20	0.13	0.01	0.03	0.52	0.02	0.28	0.04	0.14
Tema Community 1	Industrial	0.27	0.15	0.01	0.03	0.22	0.01	0.56	0.05	0.10
BLANK	ddH_2_O	0.00	0.00	−0.01	0.00	0.00	0.00	0.00	0.00	0.00
STANDARD	Standard control (Reference)	0.04	0.04	0.04	0.04	0.04	0.04	0.04	0.04	0.04

** ddH_2_O = double distilled water, ppm = parts per million, Blank = standard control, Standard = reference control, B: Boron, Ba: Barium, Cr: Chromium, Cu: Copper, Mn: Manganese, Pb: Lead, Sr: Strontium, Ti: Titanium, Zn: Zinc

**Table 5:** Univariate Analysis to Determine the Relationship Between Heavy Metal and Resistant Status in *An. gambiae* s.l.

Insecticide	Heavy metal	Coefficient	95% CI	P value
Chlorfenapyr	Mn	6.85	3.22–10.49	**0.01**
	Sr	2.24	−3.49–7.96	0.34
	Ba	− .93	−6.09 4.24	0.66
Clothianidin	Mn	− .32	−7.73 7.09	0.92
	Pb	120.44	11.39 229.49	**0.04**
	Cu	144.59	66.86 222.33	**0.01**
	Sr	1.74	−12.85 16.33	0.78
	Zn	19.23	−1.66 40.12	0.06
	Ba	−3.08	−8.45 2.29	0.20
	Ti	45.61	3.67 87.54	**0.04**
Pirimiphos-metyl	Mn	− .045	−7.76 7.67	0.99
	Pb	50.44	−206.6941 307.58	0.65
	Cu	16.25	−353.4885 385.98	0.92
	Sr	−2.89	−42.95687 37.17	0.87
	Zn	8.53	−16.02987 33.08	0.43
	Ba	−3.08	−8.449471 2.29	0.20
	Ti	17.64	−74.90112 110.19	0.66

CI: confidence interval

## Data Availability

The datasets generated during and/or analysed during the current study are available from the corresponding author on reasonable request.
